# Investigación y explotación comercial en la bacteriología: Silvio Dessy y el Instituto Biológico Argentino entre 1908 y 1947

**DOI:** 10.1590/S0104-59702026000100003

**Published:** 2026-03-02

**Authors:** Nicolás Facundo Rojas

**Affiliations:** i Becario doctoral, Consejo Nacional de Investigaciones Científicas y Técnicas. Buenos Aires – Argentina nicolasfac.95@gmail.com

**Keywords:** Bacteriología, Agentes terapéuticos, Argentina, Silvio Dessy (1869-1951), Instituto Biológico Argentino, Bacteriology, Therapeutic agents, Argentina, Silvio Dessy (1869-1951), Argentine Biological Institute

## Abstract

Este trabajo analiza la trayectoria de Silvio Dessy, un médico italiano radicado en Argentina en 1898, para problematizar el desarrollo de carreras profesionales ligadas a la ciencia por fuera del campo académico. Este médico combinó su actuación en un espacio de investigación pública con la explotación comercial de productos terapéuticos, consolidada con la creación en 1908 del Instituto Biológico Argentino, una empresa productora de sueros y vacunas. A partir del caso de Dessy, profundizamos sobre el modo en que, durante los inicios de la bacteriología en Argentina, el desarrollo de la ciencia no estaba ligada únicamente a la investigación pública, sino que existían oportunidades para llevar adelante iniciativas privadas.

## Abstract (en)

This paper analyzes the career of Silvio Dessy, an Italian physician who immigrated to Argentina in 1898, to explore the development of science-related professional careers outside the academic field. Dessy combined his work in public research with the commercial exploitation of therapeutic products, culminating in the creation of the Argentine Biological Institute in 1908, a company dedicated to the production of serum and vaccines. Based on Dessy’s case, we investigate how, during the early days of bacteriology in Argentina, the development of science was not exclusively linked to public research but also provided opportunities to pursue private initiatives.

En el cambio del siglo XIX al XX, el desarrollo de la anatomía patológica, la parasitología, la cirugía, la inmunología, la bacteriología, entre otras, implicaron una profunda transformación de la medicina, estableciendo nuevos paradigmas cognitivos y consolidando la centralidad de las prácticas de laboratorio (Buschini, 2014; Bynum, 2006; Souza, Hurtado, 2010; Zabala, 2010). La adopción de la bacteriología, en particular, modificó el modo de abordar las enfermedades infecciosas, tanto en la dimensión institucional como en la cognitiva, a partir de las nuevas teorías, terapias y políticas sanitarias enfocadas en la intervención sobre las mismas. Este despliegue de los conocimientos bacteriológicos reconfiguró el espacio sanitario local, habilitando la posibilidad de desarrollar carreras profesionales ligadas a la práctica científica y la gestión gubernamental, así como también iniciativas de comercialización de vacunas y sueros, elaborados a partir de las nuevas técnicas (Rojas, Zabala, 2023; Zabala, Rojas, 2021, 2024).

En este trabajo nos concentraremos en la trayectoria del médico italiano Silvio Dessy, quien en 1898 se radicó en el país en el marco de este proceso y, tras desempeñarse en distintos laboratorios públicos, fundó en 1908 el Instituto Biológico Argentino (IBA), una empresa que fabricaba y comercializaba sueros y vacunas. A partir de este análisis, pretendemos dar cuenta de una de las formas que adoptó la profesión científica en los inicios de la institucionalización de la bacteriología en Argentina, que combinó la investigación con la comercialización de productos biológicos. Sin embargo, a diferencia de otras figuras relevantes del período, la trayectoria del italiano trascendió el espacio en el que el reconocimiento depende de la aprobación de pares académicos y los recursos provienen fundamentalmente del Estado. Dessy, en cambio, desarrolló un perfil profesional alternativo fuertemente ligado al ámbito comercial y, en menor medida, al académico, distanciándose por ello de figuras como Ángel Roffo, Bernardo Houssay, Salvador Mazza o Rudolph Kraus, entre otros, cuyas carreras laborales estuvieron ligadas principalmente a la investigación en instituciones públicas (Buch, 2006; Buschini, Zabala, 2015; Cavalcanti, 2013b).

Siguiendo este eje, el trabajo articula dos problemas abordados por la historiografía y la sociología de la ciencia y la tecnología recientes, que refieren, en primer lugar, a la relación entre los patrones de conformación de carreras laborales dedicadas a la ciencia, así como a las formas que ha tomado la institucionalización incipiente de las disciplinas científicas (Cueto, 1994; Cueto, Silva, 2020; Edler, 2010; Hurtado, Fernández, 2013; Jogas Jr., 2017; Löwy, 2006; Stepan, 1976; Vessuri, 2007). Estas investigaciones se han concentrado en perfiles de científicos que hicieron efectiva una carrera laboral en el escenario de inestabilidad cognitiva e institucional que caracterizó el período, y, sobre todo, en figuras que posteriormente tendrán una importancia clave en el desarrollo de las instituciones de sus respectivos países. Para el caso argentino, por ejemplo, se ha investigado cómo Bernardo Houssay, Rudolph Kraus o Ángel Roffo se transformaron en actores relevantes de sus respectivos campos disciplinares (Buch, 2006; Buschini, Zabala, 2015; Cavalcanti, 2013a). Sin embargo, el caso de Dessy responde a un tipo de trayectoria escasamente tratada en la historiografía local, ya que pone de relieve que las oportunidades ofrecidas por la bacteriología desbordaban el espacio de la investigación pública, y que el desarrollo de una carrera efectiva podía responder tanto a las demandas de la academia como a las del mercado empresarial.

Un segundo problema que nos interesa discutir en este trabajo está relacionado con las experiencias de desarrollos productivos por parte de científicos, al que la historiografía reciente ha prestado atención. En este sentido, un conjunto de investigaciones ha dado cuenta de la trayectoria de científicos que se orientaron a la actividad comercial, ligada fundamentalmente a la búsqueda de beneficio económico, especialmente en el marco del despliegue global y local de un incipiente mercado terapéutico que abarcaba tanto productos de origen químico como biológico (Dutfield, 2020; Galambos, Sewell, 1997; García, 2020). Como han mostrado esos trabajos, en el cambio de siglo esta industria tuvo un patrón de reproducción marcado por el fuerte dinamismo que daba la creación de nuevas firmas privadas, en buena medida por cuenta de médicos, farmacéuticos, veterinarios y químicos que se destacaron por sus habilidades empresariales. Entre los que se han investigado recientemente, cabe mencionar a Emil Von Behring, fundador de Behringwerke (1904), empresa a través de la cual comercializaba su suero antidiftérico, a los españoles Joaquín Ravetllat y Ramón Pla, creadores del Instituto Ravetllat-Pla (1919), los colombianos Bernardo Samper y Jorge Martínez, iniciadores del Laboratorio Samper Martínez (1917), entre otros (Cavalcanti, Sá, 2017; García, 2020; Hüntelmann, 2007; Lugo-Márquez, 2013). Todos estos casos son paradigmáticos de una apuesta centrada en la elaboración y venta de sueros y vacunas, que desde fines del siglo XIX tenían una importante valoración científica y económica, ya que eran el símbolo de la “revolución terapéutica” asociada a la bacteriología (Gradmann, Simon, 2010).

En Buenos Aires, de hecho, se conformó tempranamente un nutrido campo de desarrollo de estos productos, donde distintas iniciativas combinaban el interés científico-técnico con el comercial, representando un vector de desarrollo paralelo al del sector farmacéutico convencional, especializado en remedios de origen químico (Campins, Pfeiffer, 2011; Sedrán, Carbonetti, 2019). Estas iniciativas siguieron, al menos, dos modalidades. Por un lado, algunas figuras ligadas al espacio científico-académico vendían ocasionalmente productos que surgían como derivado de sus investigaciones, como en el caso de la “Hipofisina Houssay” de Bernardo Houssay (Buch, 2006), y la “Antitosina Kraus” de Rudolph Kraus (Cavalcanti, 2013b), cuya circulación solía tener escasa repercusión e impacto entre sus pares. Asimismo, también se conformaron empresas comerciales como el Laboratorio de Antitoxinas, fundado por el médico Julio Méndez, que tuvieron una trayectoria dinámica y sostenida en el tiempo (Zabala, Rojas, 2022b). El IBA puede ubicarse en esta segunda modalidad, tanto por su sostenibilidad en el tiempo, ya que la empresa aún existe y se enfoca en el mismo rubro, y por su receptividad a la incorporación de novedades terapéuticas, dos factores que analizaremos más adelante.

Retomando estos antecedentes, en el trabajo buscamos analizar las estrategias desplegadas por Dessy para sortear la tensión que supone mantenerse en un espacio de frontera entre la profesión científica, que tiende hacia la autonomía y es motivada por la valoración otorgada por los pares, y el desarrollo de un emprendimiento comercial. Para ello, nos concentraremos en las actividades de Dessy en los tres espacios institucionales en los que se desempeñó a lo largo de su carrera, el Instituto de Higiene Experimental de la Dirección General de Salubridad Pública (IHE) (1898-1902), el Laboratorio Central del Hospital de Clínicas de la Universidad de Buenos Aires (1902-1907), y el Laboratorio Micrográfico del Hospital Italiano (1907-1947), y en el modo en que estas se articularon con la creación y gestión del IBA (1908). Partiendo del supuesto de que la investigación científica es un proceso que se encuentra atravesado por diversos aspectos sociales, institucionales, cognitivos y políticos (Knorr-Cetina, 1982; Löwy, 1994; Whitley, 2012), proponemos que las acciones de Dessy pueden comprenderse en relación con las lógicas particulares que dominan cada espacio, que orientaron sus decisiones al imponer reglas de juego o marcos de significado. Esta idea remite a debates clásicos dentro de las ciencias sociales, y principalmente al concepto de “estrategia”, entendido como el ajuste de las disposiciones subjetivas de los actores a las condiciones objetivas de un campo especifico (Bourdieu, 2007), y al que también hemos denominado “racionalidad” en un trabajo reciente (Zabala, Rojas, 2022a). Con esta herramienta de análisis buscamos mostrar cómo las acciones de Dessy fueron guiadas por las expectativas propias de cada espacio. Por ejemplo, el espacio científico-técnico, atravesado por el imperativo de la producción de saberes novedosos, supone generar conocimiento relevante para obtener reconocimiento de los pares, ya sea investigando una enfermedad, planteando una nueva teoría o describiendo una bacteria. En el espacio comercial, en cambio, el beneficio económico es obtenido a partir de demostrar que un producto (los sueros y vacunas elaborados por el IBA) es efectivo, lo que se deriva a su vez de la valoración técnica-científica otorgada por los usuarios (los médicos que utilizaban los productos del IBA).

En términos metodológicos, nos basamos en diversas fuentes documentales, tales como trabajos científicos, manuales, memorias institucionales y balances del IBA. Una de nuestras principales fuentes es la autobiografía de Dessy, titulada *Mi vida americana*, publicada en 1944, en la que hace un repaso por su carrera científica desde su llegada a la Argentina. Por otro lado, nuestro análisis también se sustenta en bibliografía secundaria de la historia de la ciencia y la medicina.

## Inicio de la carrera de Dessy en la Argentina: dirección del Instituto de Higiene Experimental de la Dirección General de Salubridad Pública entre 1898 y 1902

El arribo de Dessy a la Argentina se produce a través de un contrato para integrar el IHE, una institución proyectada en 1897 por el gobierno de la provincia de Buenos Aires como dependencia de su Dirección General de Salubridad Pública. De acuerdo con este proyecto, la principal función del IHE consistiría en estudiar las enfermedades infecciosas locales y proveer de sueros y vacunas a los hospitales bajo su jurisdicción (Argentina, 1897). Ese mismo año, el gobernador de la provincia de Buenos Aires, Guillermo Udaondo, le encargó al bacteriólogo Giuseppe Sanarelli, director del Instituto de Higiene de la Universidad de la República de Uruguay, que contactara a un bacteriólogo italiano para que eventualmente dirigiera el IHE en Argentina (Argentina, 1897; Dessy, 1944). Sanarelli propuso que Ferruccio Mercanti, uno de sus ayudantes, se ocupara de este puesto en Argentina. Mercanti aceptó la tarea, pero solicitó la contratación de un subjefe italiano, cuya elección quedó en manos de Maurizio Bufalini, catedrático de farmacología en la Universidad de Florencia, el cual llamó a un concurso privado del cual Dessy (1944) resultó ganador.

Dessy arribó a la Argentina en 1898. A su llegada, Mercanti ya se encontraba a cargo de las instalaciones del IHE, que formaba parte de la Sección IV de la Dirección General de Salubridad Pública, dirigida por Ángel Arce Peñalva. El IHE, inicialmente, fue emplazado en una casa de madera en el bosque de la ciudad de La Plata, cercano a otras instituciones científicas, como el Observatorio Astronómico y el Museo de Ciencias Naturales, y contaba con un hospital de animales de experimentación (Rojas, 2024). Al año siguiente, en 1899, Mercanti renunció a la dirección para radicarse en Florencia, y la dirección del IHE quedó en manos de Dessy. Para ocupar el puesto de subdirector, este último reclutó a Fernando Malenchini, primer ayudante en el Instituto de Anatomía Patológica del Hospital Santa María Novella, dirigido por Guido Banti, y en el que Dessy había sido segundo ayudante. Malenchini llegó al país en 1900 (Dessy, 1944).

## El desarrollo de una incipiente carrera científica: investigación sobre la tuberculosis y los *Actinomyces*

En el IHE, en cuyo cargo se mantuvo hasta 1902, Dessy continuó los estudios sobre la actinomicosis humana que había comenzado durante su formación en Italia, y de la actinomicosis en animales (Richelete, 1908). Los *Actinomyces* son organismos cuya clasificación a fines del siglo XIX era ambigua, hallándose atravesada por debates acerca de su morfología, y también sobre su naturaleza, dado que algunos bacteriólogos sostenían que se trataba de hongos, en tanto otros los definían como una bacteria. Asimismo, contribuyendo a su ambigüedad, los *Actinomyces* se encontraban vinculados a la patogenia de la tuberculosis, como así también a cuadros conocidos como actinomicosis, una enfermedad zoonótica que producía, en humanos y animales, nódulos en la boca o los pulmones. De este modo, al tratarse las dos de enfermedades zoonóticas, y basándose solo en la descripción clínica, un cuadro de tuberculosis podía ser producido tanto por un actinomiceto como por el bacilo de Koch, debido a la similitud de sus manifestaciones patológicas (nódulos localizados en los pulmones u otros tejidos, “granos negros”, entre otros) (Rossi Doria, 1891). En términos epidemiológicos, el italiano consideraba que los *Actinomyce*s tenían una amplia distribución en la provincia, por lo que el número de infectados era siempre creciente para el caso de la tuberculosis y desconocido en el caso de la actinomicosis. Bajo esta interpretación, este género podía ser causa tanto de la tuberculosis animal como humana, y solo los estudios bacteriológicos podían definir la auténtica etiología en cada caso. De hecho, Dessy realizó estudios que definieron tentativamente la prevalencia de la actinomicosis en el ganado de la provincia (Dessy, 1944, 1901a, 1901b; Malenchini, 1901).

Por otro lado, si bien las expectativas surgidas en torno a la actinomicosis tenían cierto correlato en el país, solo había sido descripta en la década de 1880 en algunas cabezas bovinas de la provincia de Buenos Aires por parte de Roberto Wernicke, director del Laboratorio de la Comisión de estudios de las enfermedades contagiosas del ganado (Rojas, 2024). Dessy, de hecho, realizó estudios que adelantaban que la enfermedad estaba ampliamente extendida en el ganado local, y por añadidura a la población humana (Richelete, 1908). En su autobiografía *Mi vida americana*, Dessy (1944) sostenía que a fines del siglo XIX el estudio de la actinomicosis en humanos era una novedad tanto en Europa como en América, y que podía representar el éxito científico para los médicos que quisieran investigarla, ya que se desconocía su prevalencia real y no existían terapéuticas eficaces para tratarla. Según esta reconstrucción posterior, el italiano afirmaba que la elección de este tema de investigación comenzó luego de que se graduara como médico en la Universidad de Turín y fuera nombrado ayudante en el Instituto de Anatomía Patológica dirigido por Guido Banti. En este instituto, al estar adjunto a un hospital, el personal contaba con una cantidad considerable de material de estudio, y diariamente se practicaban autopsias, tinciones y preparados microscópicos. Durante una de estas necropsias, Dessy afirma haber aislado por primera vez un *Actinomyces*, a partir de los nódulos pulmonares de un niño fallecido en el hospital, cuyo caso se había diagnosticado como tuberculosis miliar. Bajo la supervisión de Banti, realizó un preparado microscópico a partir del nódulo, cuyas características eran distintas a los nódulos tuberculosos, dado que no presentaba reblandecimientos en su zona central ni poseía el color característico. Luego, practicó una tinción, determinando que se trataba de un microorganismo similar al bacilo de Koch, pero con características peculiares, y “cuya identificación me era imposible por tratarse de cosa absolutamente nueva para mí” (Dessy, 1944, p.31).

De hecho, al presentarle a Banti su hallazgo, este le señaló la escasez de estudios sobre un microorganismo con características similares, que consideró un hongo del tipo *Cladothrix* – perteneciente al género *Actinomyces.* A pesar de esta supuesta excepcionalidad, Dessy (1944) señala que estos trabajos no fueron publicados, constituyendo el preludio de una investigación que seguiría en el IHE. En cambio, durante esta etapa, publicó distintos artículos de descripción de otros cuadros infecciosos, principalmente en revistas italianas.

Al viajar a la Argentina, Dessy (1944) llevó los cultivos de *Actinomyces* aislados en el instituto de Banti. Este hecho en sí mismo suponía un desafío técnico dadas las características del viaje. Antes de partir almacenó las bacterias en frascos de vidrio, junto a otros cultivos, y las trasladó en el interior de una valija durante el cruce oceánico y el desplazamiento hacia La Plata. Una vez arribado al país, continuó con la investigación sobre el microorganismo, practicando la producción experimental de la enfermedad en chanchitos de la India. A través de estos estudios, determinó que el niño autopsiado en Florencia había fallecido por un cuadro de pneumonitis actinomicótica lobular, producida por un actinomiceto *Streptothrichica* (y no *Cladothrix*, como inicialmente fue sugerido por Banti). Estas experiencias fueron publicadas luego en los *Anales de la Dirección General de Salubridad Pública*, el órgano de comunicación de la agencia de la cual dependía el IHE (Dessy, 1901b; Marotta, 1904).

Posteriormente, una vez que Dessy dejó la dirección, en 1902, las investigaciones sobre la actinomicosis no adquirieron continuidad en el IHE. Malenchini, al quedar a cargo, orientó los estudios a la fiebre tifoidea (Sociedad…, 1912).

## Articulación entre la racionalidad sanitaria y la comercial: vacunas y sueros

Como hemos señalado, en el IHE Dessy buscó ligar sus investigaciones con problemas sanitarios ya establecidos. De este modo, sus trabajos se orientaron hacia el estudio de la difteria, el carbunclo y la tuberculosis, un conjunto de enfermedades que las autoridades buscaban erradicar o mitigar desde mediados del siglo XIX. Dado el contacto estrecho que el personal del IHE tenía con los círculos de veterinarios de la Escuela de Veterinaria de Santa Catalina de la Universidad Nacional de La Plata, Dessy extendió sus estudios a algunas enfermedades veterinarias y zoonóticas, además de la tuberculosis y la actinomicosis bovina, como la “lombriz de las ovejas” y la “mancha bovina”. En primer lugar, con estos estudios se buscaba dar cuenta de la magnitud de la extensión de estas enfermedades en el ganado de la provincia. Por otro lado, diferenciar los casos de actinomicosis en el ganado, de los casos de tuberculosis bovina, a través de exámenes bacteriológicos, ya que se solían confundir debido a la similitud de las manifestaciones clínicas (Dessy, 1901c, 1944).

Ahora bien, esta estrategia científica ligada a los problemas sanitarios también se tradujo en una conexión con el interés comercial que marcaría la carrera de Dessy, dado que utilizó los estudios sobre enfermedades como marco para ensayar productos terapéuticos que comenzó a desarrollar al llegar a la Argentina. Además de elaborar sueros para tratar casi todas las enfermedades que se estudiaban en el IHE, Dessy inició la producción de tuberculina, que luego fue aceptada como un método estándar para detectar la tuberculosis en el ganado de los tambos provinciales, y a la que solo estaban dedicados la Oficina Sanitaria del DNH y el Instituto Bacteriológico de la Sociedad Rural (Zabala, Rojas, 2022b). Posteriormente, también prescribió este producto, que fue conocido como “Tuberculina Dessy”, para tratar la tuberculosis y la actinomicosis en humanos, ya que era elaborado a partir de una combinación de cultivos del bacilo de Koch y del *Actinomyces* traído desde Italia (Caras y Caretas, 1899; Dessy, 1944, 1901a, 1901b).

Asimismo, durante el brote de peste bubónica ocurrido en 1901, para el cual fue comisionado por el gobierno provincial para prestar atención médica, Dessy modificó el procedimiento de elaboración de la vacuna Lustig-Galeotti, desarrollada para tratar la peste y basada en la utilización del núcleo de las bacterias, con el fin de obtener una terapéutica para dicha enfermedad. Según Dessy, su vacuna contra la peste poseía mayor valor curativo porque el cultivo a partir del cual se obtenía contaba con una virulencia exaltada. Además, a diferencia de la vacuna de Lustig-Galeotti, en cuyo procedimiento se solía prestar poca importancia a la contaminación por fragmentos del resto del cuerpo bacteriano, Dessy (1901a) enfatizaba que la efectividad de su vacuna residía en la importancia dada a la obtención del núcleo en su máxima pureza. Este fue el primer ensayo de una vacuna que, desde el punto de vista del italiano, resultaba más efectiva que la original, y que luego adaptaría a la fiebre tifoidea y la gonorrea en el Laboratorio Central y el Laboratorio Micrográfico. Posteriormente, al crear el IBA, estas vacunas constituyeron la base de productos ofertados.

## Interacciones científicas y técnicas entre la Dirección del Laboratorio Central y el Laboratorio Micrográfico

El vínculo con la investigación científica – y el despliegue de acciones para lograr el reconocimiento de ese campo – fue una marca permanente en la carrera de Dessy, en los distintos ámbitos en los que se desempeñó, antes y durante la existencia del IBA. Entre 1902 y 1907, Dessy ejerció la dirección del Laboratorio Central del Hospital de Clínicas. Durante esos años, su interés en la elaboración de productos biológicos, la tuberculosis y los *Actinomyces* se fortaleció, en el marco de las tareas de exámenes que demandaban los servicios del Hospital. A partir de 1907 asumió la dirección del Hospital Micrográfico, creado ese año en el Hospital Italiano, cuya función era practicar los exámenes bacteriológicos de la institución (Rezzónico, 1985). Al frente de este laboratorio, cuya dirección se extiende hasta su jubilación en 1947, sus intereses científicos se desplazaron hacia otros objetos de investigación, vinculados a la búsqueda y ensayo de nuevos fármacos. En conjunto con estas actividades, Dessy buscó la permanente legitimación de su actividad científica fundando dos revistas, la *Revista Sudamericana de Ciencias Médicas y Farmacéuticas* (1903) y la *Revista Sudamericana de Endocrinología, Inmunología, Quimioterapia* (1918), donde se publicaban sus trabajos o los de médicos que valoraban positivamente sus productos curativos.

Por ejemplo, en el Laboratorio Central, los estudios sobre los *Actinomyces* fueron el objeto de conocimiento principal, tanto del propio Dessy como de los estudiantes que hacían sus prácticas en este espacio. De hecho, en este laboratorio se describió el primer caso de actinomicosis humana en la Argentina, adjudicado al médico Daniel Cranwell y al estudiante Armando Marotta (Cometto, 1905). Asimismo, durante estos años, Dessy utilizó su procedimiento con potasa para obtener una vacuna nucleoproteica contra la fiebre tifoidea, que comenzó a circular y fue ensayada en diversos servicios médicos de la ciudad.

Al frente del Laboratorio Micrográfico, Dessy continuó inicialmente con las tareas que había llevado adelante en el Laboratorio Central. Durante la primera década de dirección terminó de ensayarse su vacuna nucleoproteica contra la fiebre tifoidea y se desarrolló y elaboró una contra la gonorrea. En cambio, los *Actinomyces* representaron un tema marginal en el marco de las actividades del Laboratorio, cuyas tareas se diversificaron y siguieron los cambios en los intereses de Dessy al crearse el IBA. Estos se centraron en la búsqueda y ensayo de nuevos productos terapéuticos, además de los biológicos, incluyendo también a los de origen químico (como el digital). Este desplazamiento fue acompañado por un abandono progresivo de la actinomicosis como objeto. En gran medida, esto se debió al surgimiento de la micología médica, que resolvía el problema de la ambigüedad en la definición de los *Actynomices* caracterizándolos como hongos patógenos. En 1926, por ejemplo, fue creada la Sección Micológica del Instituto Bacteriológico del DNH, cuyo programa consistía en el estudio de la actinomicosis como una enfermedad propiamente humana, diferenciándose de la veterinaria y de los debates sobre la transmisibilidad y bacteriología de la tuberculosis (Sordelli, 1941). En el Laboratorio Micrográfico, en cambio, los escasos trabajos sobre la enfermedad practicados durante estos años siguieron vinculándola a los debates sobre su morfología y el problema de diferenciarla de la tuberculosis (Dessy, 1944).

## Actividades científicas y técnicas en el Laboratorio Central entre 1902 y 1907

El Laboratorio Central se fundó y fue puesto bajo la dirección de Dessy en 1902, quien se mantuvo al frente durante los siguientes cinco años. Esta iniciativa surgió de Pascual Palma, director del Hospital de Clínicas y Catedrático de Clínica Quirúrgica, quien contactó al italiano para que dirigiera un laboratorio que centralizara los estudios clínicos y microscópicos de los servicios de la institución (Dessy, 1944).

Al fundarse el laboratorio, los trabajos de Dessy representaron una continuidad con sus estudios llevados a cabo en el IHE. Además de realizar los diagnósticos biológicos y estudios anatomopatológicos demandados por las cátedras, y por sugerencia de Gregorio Chaves y Antonio Gandolfo (titulares de las cátedras de Clínica Médica y de Clínica Quirúrgica, respectivamente), se inició la búsqueda de un caso de actinomicosis humana como uno de los principales objetivos de investigación (Dessy, 1944). Por ello las tareas de Dessy y sus ayudantes se centraron en la caracterización y estudio de los *Actynomices*, sin dejar de lado otros tipos de estudios bacteriológicos o clínicos.

A pesar de las investigaciones hechas en el IHE, aún no se había registrado un caso autóctono de actinomicosis humana. Cumpliendo con las expectativas y los supuestos epidemiológicos de Dessy acerca de su amplia extensión en el país, la enfermedad fue rápidamente hallada entre los pacientes del Hospital. Aunque el italiano tuvo parte activa en este hallazgo, no ocupó un lugar central en la narrativa de cómo la enfermedad fue descripta por primera vez. En este proceso que se inició con la descripción de siete casos estudiados en el Laboratorio Central, se colocó a sus ayudantes u otros médicos como los principales descubridores. Esta investigación fue publicada en 1903 en la *Revista Sudamericana de Ciencias Médicas*, fundada por Dessy, en un trabajo firmado por Daniel Cranwell, cirujano y profesor sustituto de la Cátedra dirigida por Gandolfo (Cometto, 1905; Cranwell, 1903; Llambías, 1904; Marotta, 1904). En paralelo, Dessy (1944) desarrolló terapéuticas como la micetonina, un suero para tratar las infecciones producidas por micetos, y una vacuna curativa contra la fiebre tifoidea, haciendo uso de su procedimiento para obtener el núcleo de la bacteria (destruyendo su protoplasma con potasa).

En cuanto a los trabajos que derivaron de su inserción en el contexto institucional de la Escuela de Medicina y el Hospital de Clínicas, entre 1902 y 1907 se publicaron 12 tesis para optar por el doctorado, en las cuales se plasmaron trabajos experimentales llevados a cabo en el Laboratorio por estudiantes. La mayoría no era dirigida por Dessy (1944), pero agradecía su apoyo técnico.

## Dos etapas de articulación entre las racionalidades científica y comercial entre 1907 y 1947

En 1907, Dessy (1944) renunció a la dirección del Laboratorio Central, para asumir la dirección del Laboratorio Micrográfico. Aunque los motivos de esta decisión no son claros, es posible que se haya debido a falta de apoyo de las autoridades, ya que los nombramientos de los directores del Laboratorio Central pasaron a ser decididos por el Consejo de la Facultad en el año de su renuncia. Asimismo, desde 1904 ya había comenzado a vincularse al Laboratorio Micrográfico, participando informalmente de su organización (Rezzónico, 1985). Su dirección al frente del mismo puede dividirse en dos etapas que dan cuenta de las distintas articulaciones entre las estrategias científicas y comerciales de Dessy, que en líneas generales confluyeron en que el Laboratorio Micrográfico se constituyera en un lugar de ensayo y desarrollo de los productos comercializados por el IBA.

La primera etapa, marcada por una mayor pretensión de articulación con el campo científico local e internacional, se sitúa entre 1907 y 1918, teniendo como hito la creación, en 1918, de la *Revista Sudamericana de Endocrinologia, Inmunologia, Quimioterapia*, sucesora de la *Revista Sudamericana de Ciencias Médicas*, que en los hechos fue boletín de comunicación de las tareas del Laboratorio. Durante este período se establecieron las actividades regulares del Laboratorio, abarcando la descripción de bacterias y la vacunoterapia, que constituyó un tema central (Dessy, 1944). Los discípulos de Dessy, por ejemplo, se centraron en el estudio y ensayo de sus vacunas nucleoproteicas contra la fiebre tifoidea y la gonorrea, así como también sobre la “tuberculina Dessy” (Grapiolo, Dessy, 1914; Maglione, 1916). Estas vacunas fueron desarrolladas por Dessy durante estos años, y luego se comenzaron a comercializar a través del IBA, junto a la vacuna antitífica. Las vacunas nucleoprotéicas eran un agente preventivo y terapéutico creado originalmente por Alejandro Lustig y Gino Galeotti para tratar la peste bubónica, cuyo principio activo radicaba en el aislamiento del núcleo bacteriano, obtenido a partir de un procedimiento de destrucción química. Si bien las vacunas Lustig-Galeotti no se hallaban presentes en el mercado local, dado que solo eran experimentales, Dessy modificó su técnica de producción, valiéndose de su dominio de las técnicas bacteriológicas, ofreciendo – de acuerdo con sus palabras – un producto de mayor pureza, debido a la ausencia de fragmentos bacterianos contaminantes (Grapiolo, Dessy, 1914). Esta pretendida superioridad de los productos del IBA por parte de Dessy también alcanzaba a los sueros, extraídos a partir de la sangre de caballos inmunizados con las nucleoproteínas, que debido a la pureza de estas contaban con un mayor número de anticuerpos específicos (Dessy, 1944). Por último, el interés en el desarrollo y ensayo de vacunas durante esta etapa contrasta con la tendencia a la pérdida de importancia de la actinomicosis y los *Actynomices* como objetos de conocimiento.

En una segunda etapa, que va desde 1918 hasta 1947, año en que Dessy deja la dirección, es posible observar una menor pretensión de relevancia científica de los trabajos realizados, que fueron publicados en tesis y principalmente en la *Revista Sudamericana de Endocrinologia, Inmunología, Quimioterapia*. En términos generales, tanto las tesis como las publicaciones de la *Revista* referían a objetos de presumiblemente escasa relevancia científica, tales como los diagnósticos demandados por los servicios del hospital, la descripción de bacterias, estudio de aguas o productos terapéuticos y químicos elaborados y comercializados por el IBA (Carabelli, 1918; Gómez, 1937; Palazzo, 1923; Spada, 1918, 1920). Al mismo tiempo, estos temas seguían el impulso dado por los propios intereses de Dessy al frente del IBA. Por ejemplo, entre 1937 y 1940, Irene Pisarro, química del Laboratorio y del IBA, llevó adelante una serie de estudios farmacodinámicos sobre el agua del lago Epecuén (provincia de Buenos Aires), cuyas sales esperaban comercializarse por la empresa (Dessy, 1944). Por otro lado, aunque a principios de la década de 1930 existió un intento por recuperar el estudio de la actinomicosis, estos no adquieren continuidad, lo que da cuenta del abandono progresivo de este objeto de estudio (Boccia, Palazzo, 1931; Grapiolo, Palazzo, 1931; Mazzin, Palazzo, 1932).

Hasta aquí, hemos puesto de manifiesto cómo Dessy sostuvo sus estrategias científicas a lo largo de más de cuatro décadas, ligadas sin duda a su origen profesional, pero sobre todo orientadas a otorgar legitimidad y sustento al desarrollo del IBA. El despliegue de estas, sin embargo, sufrió diferentes modificaciones, condicionadas tanto por los dos contextos institucionales en los que se desempeñó, como por el inicio de su actividad empresarial. En el siguiente apartado analizaremos el caso de esta empresa.

## El IBA entre 1908 y 1947: desarrollo de una iniciativa comercial

El IBA fue una iniciativa comercial fundada por Dessy en 1908, que representó su principal proyecto en el país. Esta empresa, cuya factoría central se emplazó en un predio de ocho hectáreas en Florencio Varela, fue creada con el objetivo de ser fundamentalmente un espacio de elaboración de agentes biológicos, orientado también a la investigación de nuevas terapéuticas (Dessy, 1944). Se trata de una empresa que, tras ser vendida por los herederos de Dessy a principios de la década de 1960, aún funciona en la actualidad bajo la denominación “Biol”. A lo largo de la primera mitad del siglo XX, el IBA dio una respuesta sostenida a la demanda local de productos para tratar las enfermedades infecciosas, junto a otros laboratorios comerciales nacionales, como el Laboratorio de Antitoxinas de Julio Méndez, o empresas internacionales, como Bayer, Hoechst-Marburg, Merck, Parke & Davis, Eli, Lilly & Co y el Instituto Seroterápico de Milán (Dessy, 1944). La empresa fue organizada retomando la experiencia de estos institutos, y específicamente del Instituto Seroterápico de Milán. Estos formaron parte de la “renovación terapéutica” ocurrida en el cambio del siglo, un nuevo paradigma mediante el cual las enfermedades infecciosas comenzaron a ser diagnosticadas y tratadas con productos de origen bacteriano, como las vacunas curativas, los sueros equinos y comprimidos hechos a partir de órganos animales (llamados “organoterapicos”). El fundamento teórico de su uso descansaba en la idea de que la inoculación de elementos orgánicos procesados podía restablecer las funciones normales (inmunológicas, endócrinas etc.) del cuerpo. Los institutos seroterápicos internacionales solían producir y comercializar una amplia gama de sueros, vacunas y comprimidos organoterapicos, y eran considerados exitosos en el plano técnico y económico (Dutfield, 2020; Zabala, Rojas, 2022b).

De este modo, la empresa se centró inicialmente en la elaboración de sueros y vacunas, productos altamente demandados por el mercado médico local, diversificando posteriormente la oferta. En este proceso, el tipo de productos ofrecidos sufrió distintos avatares, que muestran la valoración que se les daba en cada etapa. Por ejemplo, en un primer momento los sueros tuvieron predominancia entre los agentes de origen bacteriano comercializados por el IBA, en tanto las vacunas comenzaron a disputar esta centralidad en la década de 1940. Esta dinámica da cuenta de cómo a través de esta empresa Dessy buscó desarrollar su carrera atendiendo de manera flexible a distintas racionalidades (científica, de atención médica y comercial). Asimismo, y fuertemente ligado a esta característica, el IBA tuvo un proceso de desarrollo similar al de otros institutos públicos, como el IB, que le permitieron mantenerse en un espacio de frontera, atendiendo a múltiples demandas, ya sea que fueran sanitarias o médicas.

Esta articulación se asociaba a la idea de que debía alcanzarse grado de producción elevado, en relación con una virtual alta demanda. Por ejemplo, se estimaba que cada tratamiento antiinfeccioso practicado en hospitales, clínicas o consultorios, y también la vacunación preventiva del ganado, requería de un número considerable de unidades de vacunas o sueros (Zabala, Rojas, 2022a). Para sostener estos niveles de producción, la institución contaba con un sistema de división del trabajo, organizado a partir de secciones, en el que se llevaban a cabo todas las fases de producción de los cultivos bacterianos, así como de su empaque, y de la inmunización de los caballos y extracción del suero. Además, la empresa contaba con establos de cría de los caballos y las cabras, en los que se inoculaban los cultivos bacterianos y se ensayaba su virulencia. Junto a estos, el IBA también disponía de secciones encargadas de la obtención de productos organoterapicos y un invernadero de plantas medicinales (Gastaldi, 1926).

Uno de los principales factores que dan cuenta de esta adaptación es que durante la primera década de funcionamiento, de acuerdo con lo que se desprende de sus balances financieros, el IBA ya contaba con un importante superávit comercial. Este se sustentó en diversos factores, pero principalmente en la amplia expansión de su oferta en el mercado local – favorecida por la coyuntura de la Primera Guerra Mundial y la escasez de terapéuticas importadas –, donde abastecía a particulares y a hospitales, la introducción y venta de algunos productos extranjeros (sueros de Berna) y la exportación hacia países vecinos (Uruguay, Paraguay, Brasil y Chile) (Instituto…, 1947). En la década 1920 y 1930, la empresa llevó adelante una sostenida campaña publicitaria en los países en los que operaba (entrega de folletos, publicidades en revistas médicas locales e internacionales, asistencia a los clientes a través de correspondencia), además de contar con una importante oficina comercial en la capital federal (Instituto…, 1934). A fines de la década de 1940, la empresa aún seguía reportando cuantiosas ganancias y renovando sus instalaciones fabriles (Instituto…, 1947).

El segundo factor de desarrollo lo encontramos en la dinámica de organización del personal técnico de los laboratorios, dado que Dessy reclutó al personal del IBA entre discípulos e investigadores que contaban con experiencia en laboratorios, ya sea que se dedicaran a los estudios bacteriológicos, químicos o bioquímicos. De este modo, la empresa se transformó en un espacio que aumentaba las posibilidades de inserción profesional de un cuerpo de especialistas, que muchas veces alternaban entre los espacios público y privado. Entre los primeros se encontraban Armando Marotta, estudiante de medicina de la Escuela de Medicina de la UBA, y aprendiz de Dessy en el Laboratorio Central, donde había identificado uno de los primeros casos de actinomicosis. Marotta fue nombrado socio de la comisión directiva de la empresa y posteriormente designado presidente (Guía…, 1947). De modo similar, otros estudiantes de medicina que pasaron por el Laboratorio Micrográfico fueron reclutados para desempeñarse en el IBA, como José Malaspina (Sección análisis), Irene Pisarro (Sección Química Biológica), Miguel Mamone (Sección Análisis Clínicos) y Eduardo Peano (Ayudante de la Sección Farmacología). En cuanto a los segundos, Dessy contrató a investigadores con experiencia, tanto del escenario local, como Alois Bachmann (antiguo director del IB del DNH), como del extranjero, entre los que se contaban el químico italiano Gerónimo Spagnol (*fellow* de la Fundación Rockefeller), la médica alemana Gerda Meyer (graduada de la Universidad de Florencia), o el ingeniero Lorenzo Banti (graduado de la Universidad de Turín e hijo de Guido Banti), entre otros (Dessy, 1944).

Estos factores aumentaron la capacidad de adaptación a los nuevos procesos de producción, ya que las diversas novedades terapéuticas que surgían en la medicina eran rápidamente elaboradas y comercializadas (Comité…, 1941). Durante los casi 40 años de funcionamiento analizados en este trabajo, el IBA se adaptó a todas las novedades del campo terapéutico, comercializando hormonas, vitaminas, insulina y antibióticos, entre otros. La producción teórica acerca de nuevas terapéuticas por parte de Dessy (1944), en cambio, fue escasa o inexistente, centrándose sobre todo en las vacunas curativas. Hacia 1913 el IBA ofertaba 20 productos, 15 de los cuales eran sueros, y 2 vacunas (los restantes eran productos antiinfecciosos de origen químico); en 1936, de un total de 88 productos, entre los que también se contaban distintos tipos de insulinas, vitaminas y productos antiinfecciosos de origen químico, 15 eran sueros y 10 vacunas. En 1941, de 224 productos comercializados por la empresa, 22 eran sueros y 28 vacunas. Para dar una idea de la importante posición del IBA en el campo local de productos biológicos, cabe resaltar que en 1913 Hoechst-Marburg, comercializadora de los productos Bayer, ofrecían 30 productos (14 sueros y 14 vacunas), y Milet y Roux, introductores de los sueros y vacunas del Instituto Pasteur, 27 productos (10 sueros y 12 vacunas). En 1941, estos habían ascendido a 176 en el caso de la primera (22 sueros y 26 vacunas), y 152 en el caso de la segunda (5 sueros y 17 vacunas) (Comité…, 1936, 1941; Dessy, 1944).

Como hemos mencionado, la oferta del IBA estuvo fuertemente ligada a distintas racionalidades. Los sueros, aislados a partir de la sangre de caballos inmunizados, solían ser utilizados como un tratamiento antiinfeccioso estándar, en conjunto con las vacunas, que consistían en cultivos bacterianos graduados y envasados que eran inoculados gradualmente en los pacientes con el fin de estimular su sistema inmune (Zabala, Rojas, 2022b). Según el italiano, sus vacunas, debido a su supuesta alta pureza, producían una reacción inmune de mayor potencia que el resto de las vacunas importadas o fabricadas en laboratorios nacionales. Además, ya que estas se utilizaban para inmunizar a los caballos a partir de los cuales se extraía el suero terapéutico, éste último poseía mayor cantidad de anticuerpos curativos (Dessy, 1944).

De modo más específico, atendiendo a esta valoración médica, Dessy elaboró y comercializó a través del IBA dos de los sueros que habían secundado la renovación seroterápica, el suero antidiftérico y el antitetánico, sin dejar de lado otros como el antineumónico (contra la neumonía), antipiógeno, antiestreptocócicos y antimeningocócicos (indicados para el tratamiento de infecciones locales, septicemias y meningitis cerebro espinales, respectivamente), así como también anti antidisentéricos (elaborado a partir de *Salmonellas*), entre otros. El IBA también comercializaba suero anticarbuncloso, indicado para el tratamiento del carbunclo humano, y antipestoso. Las enfermedades para las cuales algunos estaban indicados eran poco frecuentes (la peste, la meningitis y el carbunclo, son las tres más representativas), y solo eran demandados por hospitales o instituciones sanitarias públicas (como la Asistencia Pública de la ciudad de Buenos Aires), que eran clientes del IBA (Instituto…, 1913). Además de estos sueros, cuyo valor curativo residía en los anticuerpos específicos que poseían, Dessy elaboró sueros normales de caprinos, extraídos de las arterias de cabras que no eran previamente inmunizadas. Según el italiano, los sueros normales, que estaban siendo ensayados en otros institutos, representaban una significativa innovación terapéutica, que curaba a partir de estimular y fortalecer el sistema inmune del infectado, facilitando así la producción de anticuerpos por parte de su organismo (Dessy, 1944).

Las vacunas nucleoprotéicas, que Dessy había elaborado desde su llegada a la Argentina, tanto en el IHE como en el Laboratorio Central y el Laboratorio Micrográfico, fueron el producto principal ofertado por el IBA. La elaboración de estos productos se centró, durante la primera década de funcionamiento de la empresa, en la tuberculina Dessy, la actinomicina, y en las vacunas nucleoprotéicas antigonocócica y antitífica, a las que luego se incorporaron las vacunas antipiógena polivalente, antigripal, antiestafilocócica y antineumocócica, desarrolladas íntegramente en sus laboratorios (Círculo…, 1930; Comité…, 1936; Dessy, 1944). A principios de la década de 1920, el IBA también comenzó a comercializar vacuna anticarbunclosa, utilizada en la prevención del ántrax en el ganado, y que era un tipo de preventivo altamente demandado entre los ganaderos locales e internacionales (República…, 1921).

La inserción de Dessy dentro del campo de la atención médica alcanzaba a múltiples instituciones, y formaba una parte importante de su proceso de elaboración y distribución de productos terapéuticos. De hecho, desde las primeras décadas del siglo Dessy conformó una red de ensayo, que vinculaba al IBA con el Laboratorio Micrográfico, con los servicios clínicos del Hospital Italiano, y con otros espacios de atención hospitalaria de la ciudad, como el Hospital de Clínicas, y de la provincia, entre los que se contaban el Hospital San Juan de Dios de La Plata. Por ejemplo, en el Servicio de Ginecología del Hospital de Clínicas, dirigido por Carlos Castaño, la nucleoproteína Dessy era considerado el producto terapéutico estándar en el tratamiento de la gonorrea y de diversas infecciones uterinas, a pesar de sus efectos colaterales (intenso dolor), superior a otras vacunas nacionales o importadas (Castaño, 1921). Castaño, que era uno de los líderes de la renovación terapéutica en los hospitales porteños, e impulsaba fuertemente el uso de agentes curativos bacterianos, proclamaba la efectividad de las vacunas de Dessy, incluso por sobre otras disponibles: “Yo he experimentado todas esas vacunas, y cuando escribí ese libro sobre vacunoterapia gonocócica, fuimos los que primero se ocuparon de estas cuestiones; estudié todas las vacunas …; he encontrado que la nucleoproteína es excelente: hoy está modificada para atenuar el intensísimo dolor que provoca en las enfermas” (p.129).

Dessy también tuvo un vínculo sostenido y por correspondencia con los médicos de países que importaban las vacunas y sueros del IBA, que solían remitir estudios o síntesis de sus experiencias con estos productos (Dessy, 1944). Esta dinámica tuvo un fuerte impulso durante las primeras décadas de funcionamiento de la empresa, y puede comprobarse en *Mi vida americana* (Dessy, 1944)*,* en la que el italiano anexó una “Nómina de algunos trabajos científicos que tratan del empleo terapéutico de productos biológicos del doctor Silvio Dessy”. Se trata de 41 artículos publicados en el período de 1907-1918 y nueve trabajos publicados entre 1918 y 1945, que excedían el ámbito argentino, dado que también reflejan experiencias de Brasil, Italia y Uruguay. La mayoría de los trabajos trata del uso de las vacunas nucleoprotéicas en el tratamiento de la gonorrea y la fiebre tifoidea, y de la tuberculina Dessy para tratar casos de actinomicosis y tuberculosis. Asimismo, los médicos extranjeros solían recibir asistencia por correspondencia acerca del uso de los productos, en ocasiones por parte del propio Dessy (Instituto…, 1913; Dessy, 1944).

## Consideraciones finales

La trayectoria de Silvio Dessy da cuenta de un modo poco explorado en el ámbito regional de llevar adelante una carrera profesional ligada a la ciencia, pero que formaba parte de las posibilidades existentes en el marco de la incorporación local de la bacteriología. Por otro lado, su trayectoria también muestra las tensiones propias de este proceso, que se expresan en el plano cognitivo y social. El primer aspecto que queremos discutir en ese sentido es de la incertidumbre de las apuestas individuales, que en este marco de inestabilidad no siempre se traducían en beneficios inmediatos. Dessy se situó en una posición de frontera, con una débil conexión con el mundo científico, orientada en función de su actividad empresarial, que fue su principal apuesta. Por ejemplo, al llegar a la Argentina, dedicó sus esfuerzos a investigar la actinomicosis, una enfermedad que, si bien desconocida, estaba fuertemente asociada a la tuberculosis y su importancia sanitaria. Sin embargo, sus aportes hechos al tema no se tradujeron en reconocimiento científico, y en cambio, tuvo una posición marginal al interior del campo académico. Esta condición fue beneficiosa para su carrera, ya que le permitió mantenerse vigente en el espacio de la investigación y la atención médica durante varias décadas. Este vínculo fue clave para obtener la valoración que los productos del IBA requerían, ya sea circulando entre otros médicos dedicados a la atención, o sirviendo como espacios de testeo (como en el caso del Laboratorio Micrográfico).

En el plano social, como resultado de esta dinámica, la trayectoria de Dessy se diferenció de otros perfiles de médicos-investigadores de la época, como Bernardo Houssay o Ángel Roffo, que ligaron sus carreras fundamentalmente al espacio académico. La historiografía reciente se ha enfocado en este tipo de perfiles, señalando que el impulso dado por el Estado fue el principal vector de desarrollo científico durante el periodo. Sin embargo, este trabajo muestra que la investigación pública no era el único ámbito en el que se podía transformar una vocación científica en una carrera laboral. El caso de Dessy pone de relieve las distintas respuestas que podían darse a las exigencias del espacio académico y empresarial, así como la flexibilidad de las actividades que podían llevarse adelante. Por ejemplo, la agenda científica del italiano fue variando a lo largo del tiempo, y hacia la década de 1930 la actinomicosis ya no ocupaba una parte central de sus intereses. En cambio, el testeo de nuevos productos, muchos de los cuales eran fabricados en el IBA, pasó a ser uno de sus principales intereses. Esto es una muestra de que la investigación respondía también a factores exógenos y que los marcos de significado eran inestables y estaban sujetos al cambio.

Siguiendo esta interpretación, entendemos que el desarrollo del IBA representó el principal posicionamiento profesional de Dessy, dado que a través de esta iniciativa introdujo productos de elaboración propia en el mercado local e internacional, obtuvo considerables ganancias económicas y logró continuidad en el tiempo. El principal factor que le permitió este posicionamiento se encuentra en la amplia recepción que tuvieron los productos del IBA entre los médicos locales y de los países a los que eran importados. Asociado a esto, el IBA respondió a los cambios de paradigma del mercado terapéutico, y su presencia en el mercado siguió estos avatares. Por ejemplo, durante las primeras décadas de funcionamiento, al centrarse en una considerable oferta de distintos tipos de vacunas y sueros, la presencia del IBA en el mercado local fue considerable, rivalizando con empresas extranjeras. Estos productos competían con otros que ya tenían presencia en el mercado local, incluso sustituyéndolos, como en el caso de la vacuna nucleoproteica anti gonocócica.

Por último, nos interesa señalar que la valoración diferencial de las vacunas y sueros del IBA tenía su fuente en el dominio de las técnicas bacteriológicas por parte de Dessy. El italiano era reconocido entre los médicos de Buenos Aires por este saber experto, que le permitió obtener agentes terapéuticos ampliamente valorados por su efectividad. Dado que existían otros productos similares en el mercado local, su éxito se basó exclusivamente en la valoración otorgada en distintos servicios médicos de Argentina y los países a los que eran exportadas, que solían publicar artículos en la *Revista Sudamericana de Endocrinología, Inmunología, Quimioterapia*, donde recopilaban estas experiencias, en todos los casos con resultados positivos.

**Figura 1:**
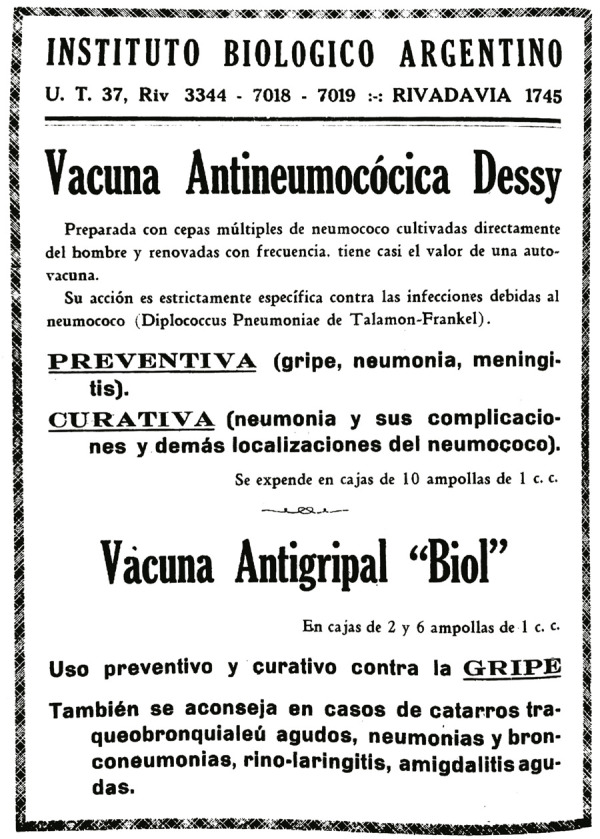
: Publicidad de las vacunas antineumocócica y antigripal del IBA publicada en *El Día Médico* de Argentina (Vacuna antineumocócica…, 1933, p.564)

**Figura 2:**
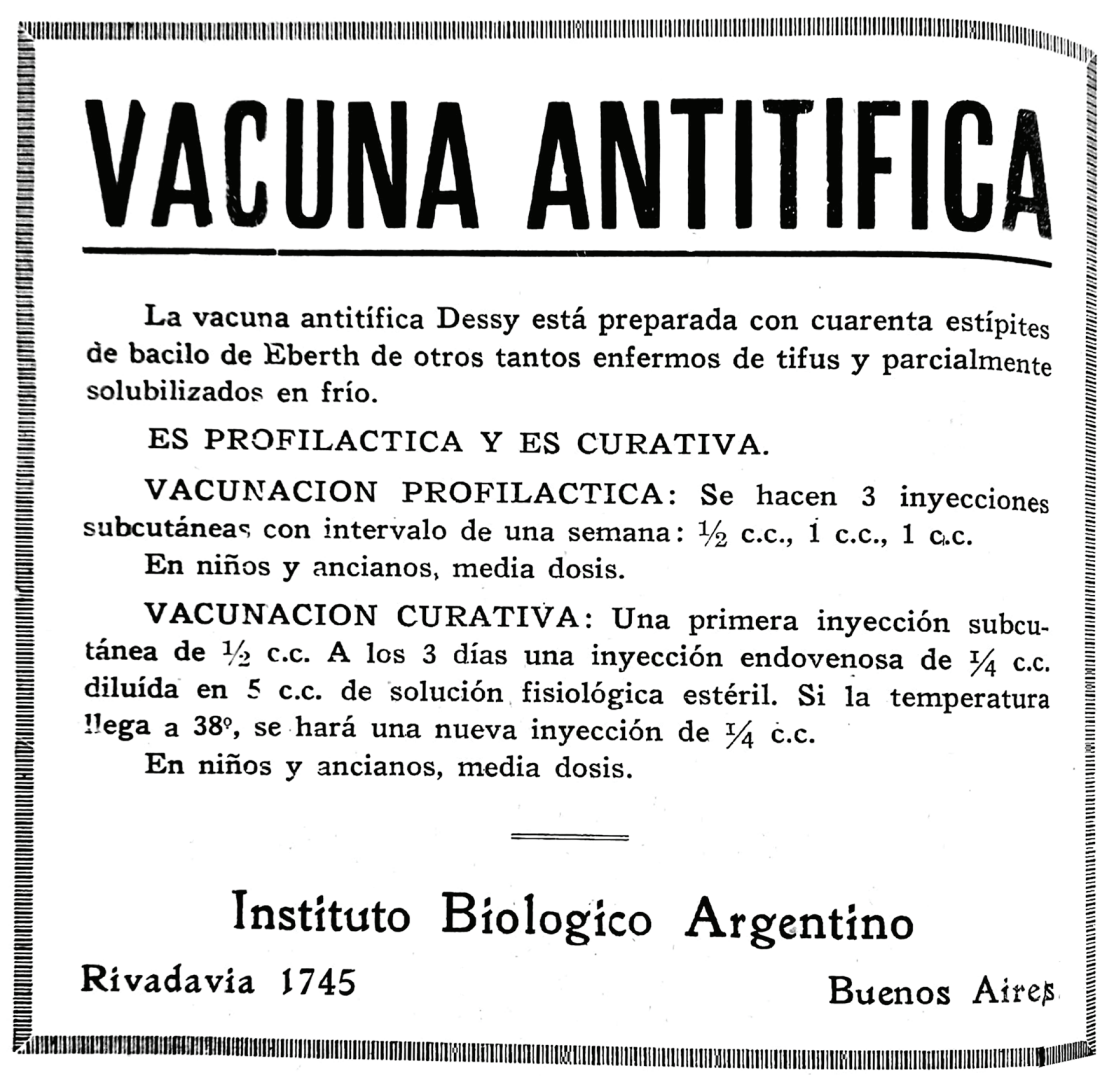
: Publicidad de la vacuna antitífica publicada en *El Día Médico* de Argentina (Vacuna antitífica, 1933, p.567)

**Figura 3:**
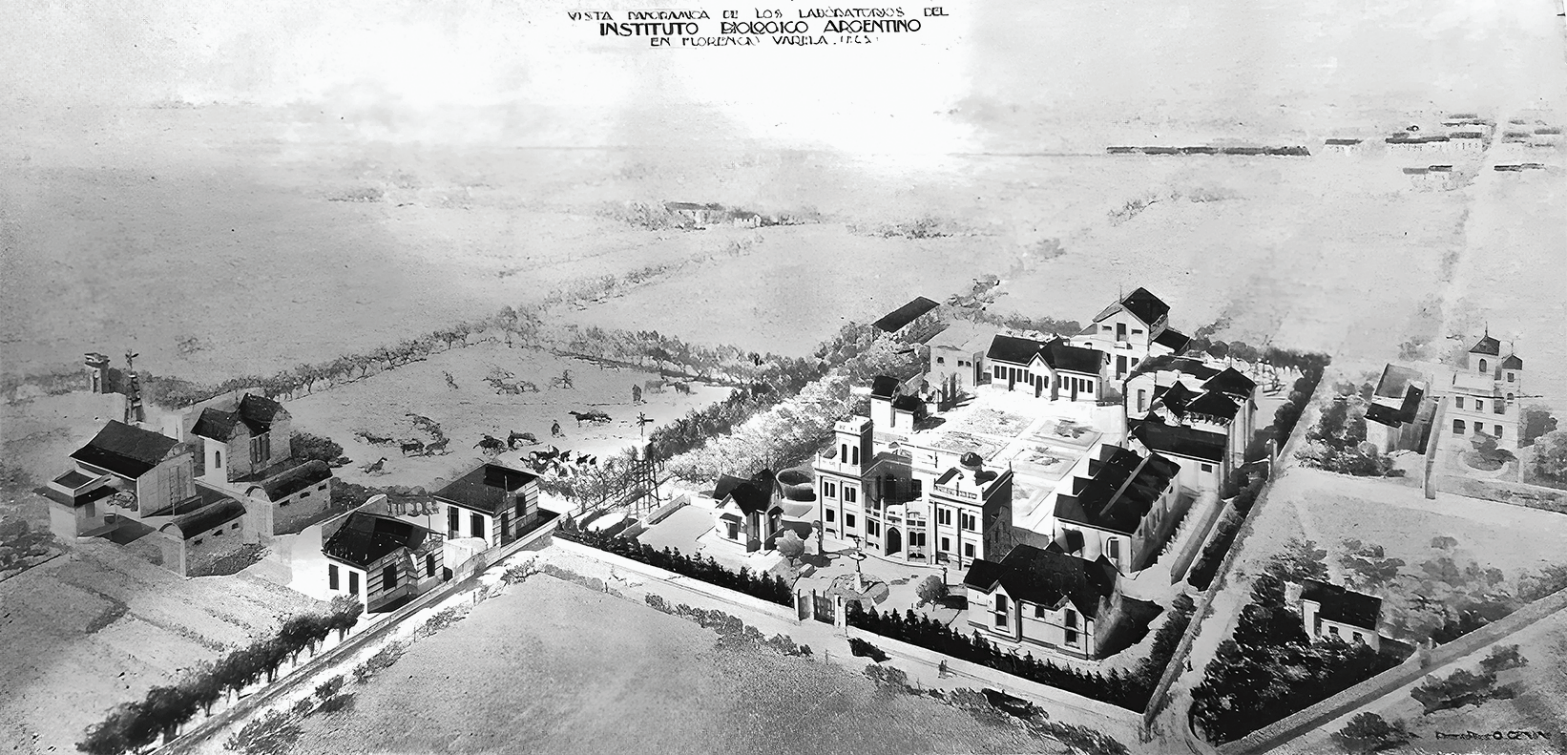
: Planta industrial del IBA en Florencio Varela, provincia de Buenos Aires (Instituto…, 1926)

**Figura 4:**
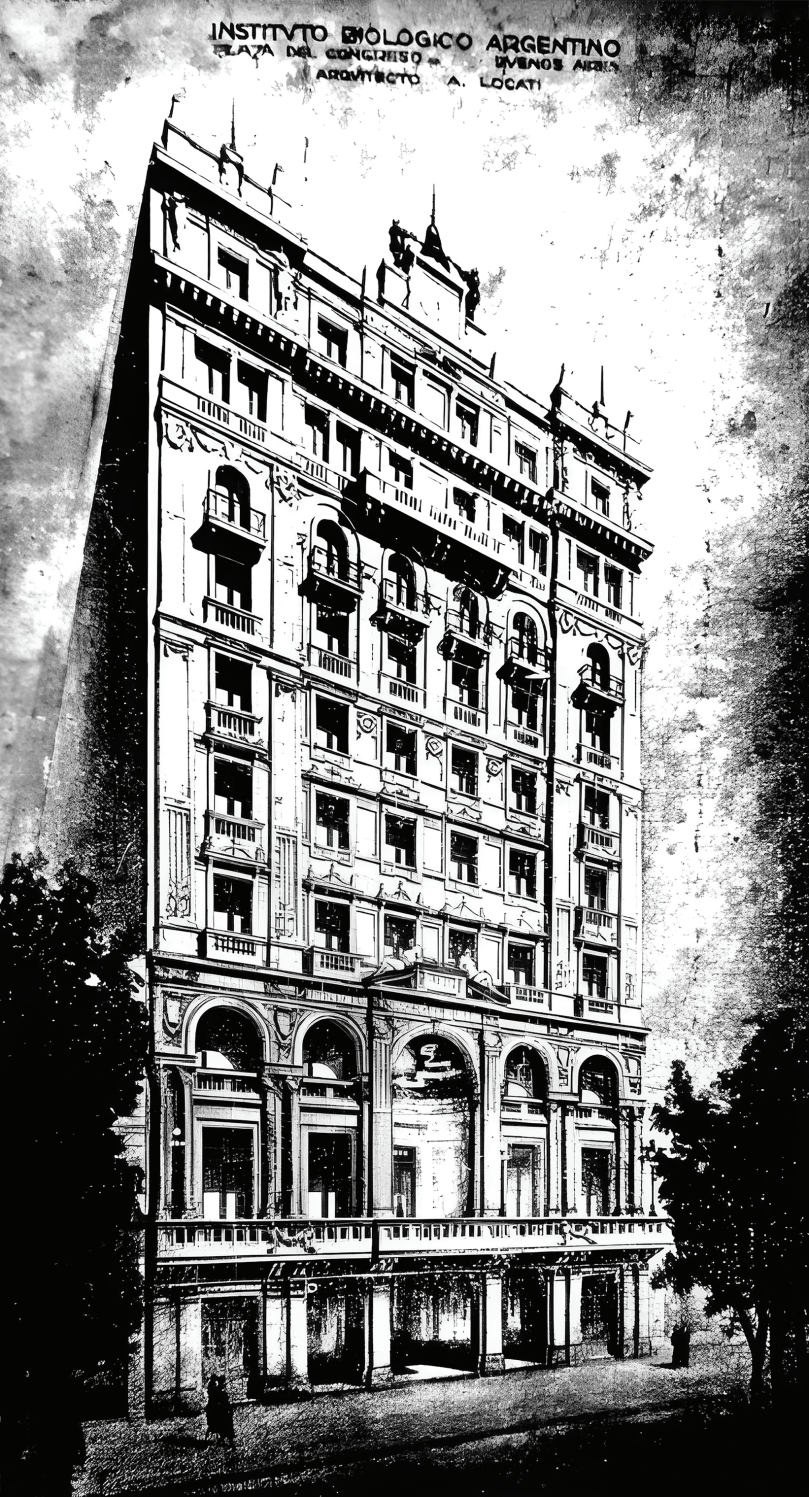
: Oficina comercial del IBA en la ciudad de Buenos Aires, frente a la Plaza Congreso (Instituto…, 1926)

## Data Availability

No están en repositorios.
